# GLP-1 receptor activated insulin secretion from pancreatic β-cells: mechanism and glucose dependence

**DOI:** 10.1111/j.1463-1326.2012.01663.x

**Published:** 2012-08-01

**Authors:** A R Meloni, M B DeYoung, C Lowe, D G Parkes

**Affiliations:** Amylin Pharmaceuticals, Inc.San Diego, CA, USA

**Keywords:** β-cell, antidiabetic drug, drug mechanism, GLP-1, insulin secretion, type 2 diabetes

## Abstract

The major goal in the treatment of type 2 diabetes mellitus is to control the hyperglycaemia characteristic of the disease. However, treatment with common therapies such as insulin or insulinotrophic sulphonylureas (SU), while effective in reducing hyperglycaemia, may impose a greater risk of hypoglycaemia, as neither therapy is self-regulated by ambient blood glucose concentrations. Hypoglycaemia has been associated with adverse physical and psychological outcomes and may contribute to negative cardiovascular events; hence minimization of hypoglycaemia risk is clinically advantageous. Stimulation of insulin secretion from pancreatic β-cells by glucagon-like peptide 1 receptor (GLP-1R) agonists is known to be glucose-dependent. GLP-1R agonists potentiate glucose-stimulated insulin secretion and have little or no activity on insulin secretion in the absence of elevated blood glucose concentrations. This ‘glucose-regulated’ activity of GLP-1R agonists makes them useful and potentially safer therapeutics for overall glucose control compared to non-regulated therapies; hyperglycaemia can be reduced with minimal hypoglycaemia. While the inherent mechanism of action of GLP-1R agonists mediates their glucose dependence, studies in rats suggest that SUs may uncouple this dependence. This hypothesis is supported by clinical studies showing that the majority of events of hypoglycaemia in patients treated with GLP-1R agonists occur in patients treated with a concomitant SU. This review aims to discuss the current understanding of the mechanisms by which GLP-1R signalling promotes insulin secretion from pancreatic β-cells via a glucose-dependent process.

## Introduction

Plasma glucose is tightly regulated by the coordinated actions of the pancreatic hormones insulin and glucagon, which act in opposition to keep plasma glucose levels constant. Patients with type 2 diabetes (T2D) often experience β-cell failure leading to reduced production and secretion of insulin. The reduced insulin secretion is compounded by increased insulin resistance, excessive glucagon-mediated release of glucose from hepatic stores, and rapid glucose influx during meals [1]. Together, the inability to maintain glucose homeostasis leads to a perpetual state of hyperglycaemia. Exogenous insulin or sulphonylurea (SU)-based therapies, while valuable in reducing hyperglycaemia, continue to act even after an ideal glucose concentration has been met and therefore pose the risk of over-correction and hypoglycaemia [2]. The symptoms of hypoglycaemia can range from trembling and weakness to poor coordination and impaired cognition. Studies of the effects of hypoglycaemia on patient quality of life (QoL) suggest that QoL is reduced in patients experiencing moderate to profound hypoglycaemia compared to patients reporting mild or no symptoms of hypoglycaemia [3]. In addition to unwanted symptoms, hypoglycaemia has been associated with QT interval prolongation, arrhythmias and other adverse cardiovascular events [4–9]. Thus, hypoglycaemia is a significant clinical concern for patients with diabetes and minimizing the risk of hypoglycaemia is important in any diabetes therapy.

The glucose-dependent T2D therapies such as glucagon-like peptide-1 receptor (GLP-1R) agonists and dipeptidyl peptidase-4 inhibitors, promote glucose control by minimizing both hyperglycaemia and hypoglycaemia. Glucagon-like peptide 1 (GLP-1; 7–36 amide), an incretin hormone secreted by intestinal L-cells in response to glucose and other ingested nutrients, induces insulin secretion via the GLP-1R in a glucose-regulated manner. While effects of GLP-1R signalling, such as suppression of glucagon secretion, slowed gastric emptying, and increased satiety, are also involved in glucose control by GLP-1R agonists, pancreatic β-cell-mediated insulin secretion is the best characterized GLP-1R activity (for additional review see Ref. [10]). The ability of GLP-1 and GLP-1R agonists to promote insulin secretion depends upon elevated blood glucose levels. As such, the activity of GLP-1 and its receptor agonists is self-limiting, ceasing when blood glucose levels fall in response to the secreted insulin. With the increasing use of GLP-1R agonists for treatment of T2D, a clarification of the current understanding of the mechanisms by which GLP-1 receptor signalling promotes insulin secretion and the dependence of this process on glucose is warranted.

## GLP-1 Receptor Agonists

The GLP-1 peptide itself is short-lived (t_1/2_ = 1.5 to 5 min) [11]. However, longer-acting, synthetic, GLP-1R agonists have been developed for clinical use and others are currently in development. The first-in-class GLP-1R agonist, exenatide [BID (twice daily)] is a 39-amino-acid peptide that is a synthetic version of exendin-4. Exenatide has 53% amino acid homology to GLP-1 and binds to the GLP-1R with similar affinity as GLP-1 (for a more detailed review see Ref. [12]). Lacking the dipeptidyl peptidase-4 (DPP-4) cleavage site, exenatide has a much longer half-life than endogenous GLP-1 [13]. A second GLP-1R agonist, liraglutide (once daily) is a GLP-1 analogue with an albumin binding, fatty acid side chain. The peptide itself has an amino acid sequence that shares 97% identity with the human GLP-1 peptide [14]. With its extensive similarity to human GLP-1, liraglutide is a substrate for DPP-4 although with a much reduced rate of cleavage compared to endogenous GLP-1 [13]. Exenatide BID, its once-weekly formulation (exenatide QW [once weekly]) and liraglutide have all been clinically demonstrated to reduce hyperglycaemia, as evidenced by a reduction in the percent of haemoglobin A1c protein that is glycated (HbA1c) in patients with T2D [15–23]. Furthermore, all three GLP-1R agonists have been shown to assist people with T2D in achieving the recommended American Diabetes Association target HbA1c goal of <53 mmol/mol (<7%) [24].

## Evidence for Glucose Dependence of GLP-1R Agonist-Stimulated Insulin Secretion

The glucose dependence of the insulin secretory activity of GLP-1R agonists has been demonstrated by a variety of *in vitro* and *in vivo* studies such that it is well-accepted by those in the field [25–33]. Early *in vitro* studies in a rat insulinoma cell line demonstrated that induction of insulin secretion by GLP-1 was glucose dependent. Insulin secretion mediated by GLP-1 (10 nM) in the absence of glucose or by the presence of 10 mM glucose alone was maximally induced by between 1.5- and 2.5-fold. However, in the presence of 10 mM glucose, GLP-1 (10 nM) maximally induced insulin secretion by approximately sixfold over baseline [33]. Similarly, in the perfused rat pancreas, GLP-1 (25 nmol/l) mediated a slight insulin secretion at basal glucose concentrations (2.8 mmol/l) but when glucose concentrations were raised to 5 mmol/l, a strong GLP-1-mediated stimulation of insulin secretion, which exceeded the effects observed with glucose alone, was observed [28]. This glucose dependence of GLP-1's insulin secretagogue function was likewise demonstrated during *in vivo* studies. Fasting healthy human subjects treated with pharmacological intravenous doses of GLP-1 (7–36 amide) exhibited no hypoglycaemia despite their fasted state [30]. Together, these data provided evidence of a requirement for glucose in the insulin-stimulatory action of GLP-1 and suggested that a threshold glucose concentration was required for GLP-1 activity.

Similar to the natural GLP-1 peptide, GLP-1R agonists such as exendin-4 have likewise been shown in animal models and humans to require glucose concentrations above basal levels to promote insulin secretion. Studies of mice conditionally expressing exendin-4 revealed that even under relatively high exendin-4 expressing conditions, fasting blood glucose levels were normal and no hypoglycaemia was observed [32].

**Figure 1:**
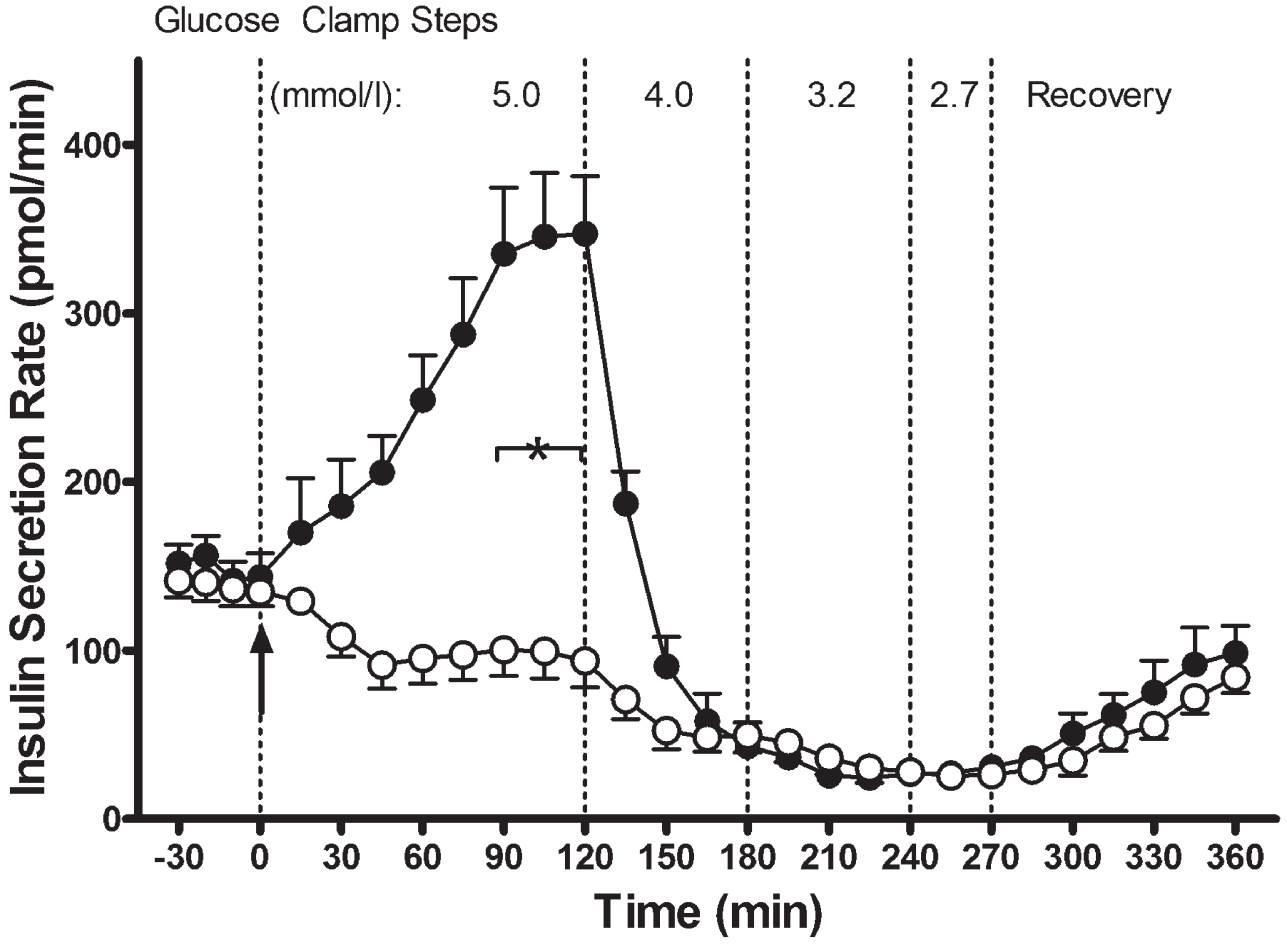
Insulin secretion. Basal timepoints from −30 to 0 min. Infusion of exenatide or placebo commenced at 0 min as indicated by arrow. From 0 to 120 min, plasma glucose was ∼5.0 mmol/l (euglycaemia). At 120–180 min, plasma glucose was ∼4.0 mmol/l (hypoglycaemia). At 180–240 min, plasma glucose was ∼3.2 mmol/l ending in nadir of ∼2.8 mmol/l (hypoglycaemia). Recovery phase from 270 to 360 min. ○, placebo treatment arm;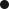, exenatide treatment arm. Data are means ± s.e.; n = 11 per treatment arm. *p < 0.05, exenatide vs. placebo during steady state of a glycaemic interval. Reproduced with permission from Degn et al. [31].

Human studies using GLP-1R agonists have provided the best support for the dependence of GLP-1R activity on glucose concentrations. As exenatide was the first widely used synthetic GLP-1R agonist in humans, much of the work examining the glucose dependence of GLP-1R-mediated insulin secretion was completed with exenatide. One study, in which exenatide or placebo was continuously infused intravenously into healthy, fasted individuals, demonstrated that subjects infused with exenatide while clamped at euglycaemic concentrations of glucose (∼5.0 mmol/l), secreted much greater amounts of insulin than the placebo-infused counterparts (∼350 pmol/min vs. ∼100 pmol/min). Demonstrating glucose dependence, insulin secretion in the same subjects infused with exenatide rapidly decreased to levels similar to the placebo counterparts when plasma glucose concentrations were dropped to hypoglycaemic levels (∼4.0 mmol/l; figure 1) [31]. Similar studies in which exenatide was administered to subjects via subcutaneous injection or intravenous infusion likewise demonstrated the glucose dependence of exenatide-mediated insulin secretion [34,35].

As might be expected of a glucose-dependent therapy, low incidences of hypoglycaemia were observed in clinical trials of exenatide QW, despite continuous exposure to the GLP-1R agonist due to extended release [16,36]. In other clinical trials, GLP-1R agonists were associated with rates of hypoglycaemia similar to those of placebo. Indeed, results from clinical studies examining the efficacy of exenatide or liraglutide as monotherapies or in combination with oral antidiabetes medications in patients with T2D have shown that, at concentrations sufficient to reduce HbA1c, no significant differences in the incidence of major [events that resulted in loss of consciousness, seizure, coma or other mental status change consistent with neuroglycopaenia which resolved after administration of glucagon or glucose or events those that required third party assistance because of severe impairment in consciousness or behaviour and had a glucose value of less than 54 mg/dl (3 mmol/l)] or minor [events that had symptoms consistent with hypoglycaemia and glucose values of less than 54 mg/dl (3 mmol/l) prior to treating the episode] hypoglycaemia compared to placebo were observed (Table 1) [15,21,37–40]. Moreover, patients using exenatide BID together with titrated insulin glargine (with or without metformin or pioglitazone or both) observed similar rates of hypoglycaemia but significantly greater reductions in HbA1c compared to patients using placebo and insulin (Table 1) [41].

**Table 1:** Percent of patients experiencing hypoglycaemia during study period

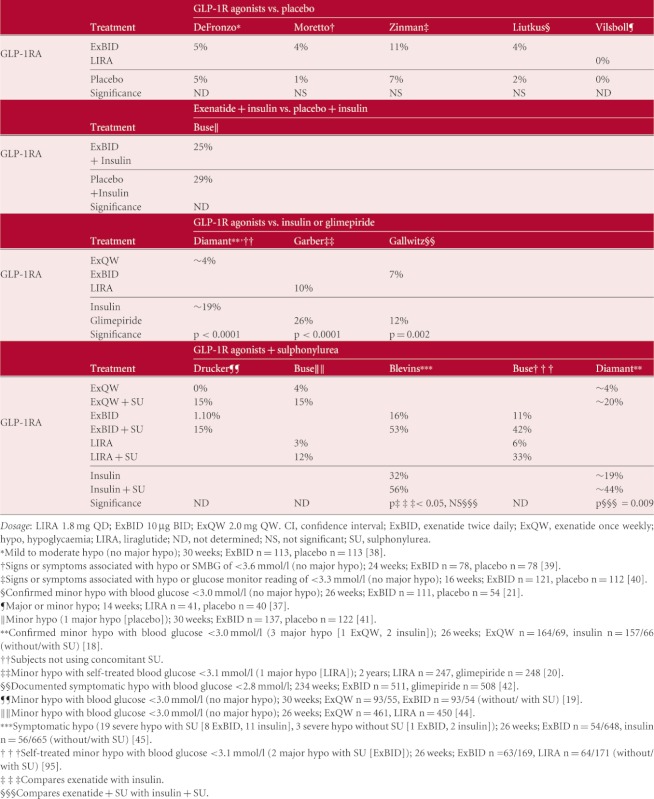

Many antidiabetes therapies have demonstrated effective reductions in HbA1c. However, the glucose-independent activity of some common therapies such as insulin glargine and glimepiride is associated with increased rates of hypoglycaemia compared to GLP-1R agonists (Table 1) [18,20,42]. Interestingly, SU-based compounds (such as glimepiride) have been observed to uncouple the glucose dependence of GLP-1R agonists and promote increased rates of hypoglycaemia in patients using GLP-1R agonists together with SU (Table 1) [17,19,43–45].

An understanding of the molecular pathways involved in insulin secretion is useful in explaining such clinical observations as the ability of GLP-1R agonists to reduce hyperglycaemia with a low likelihood of hypoglycaemia. Detailed mechanistic studies have shown that insulin secretion stimulated by high glucose concentrations (or SU use) and augmented by GLP-1R agonists, occurs via common and inter-related molecular pathways, which will be subsequently described.

## Glucose-Induced Insulin Secretion from β-Cells

### Glucose Sensing

Glucose itself plays a primary role in mediating insulin secretion. Pancreatic β-cells initially sense elevated extracellular glucose and allow its cellular entry through the GLUT facilitative glucose transporters [46]. Phosphorylation of the transported glucose promotes the conversion of glucose into pyruvate, which is then shuttled to the mitochondria to participate in the tricarboxylic acid cycle [47,48]. This process of transporting extracellular glucose into the pancreatic β-cell and its conversion into ATP leads to increases in the cellular ATP/ADP ratio (increases in ATP at the expense of ADP) and promotes the initial triggering event for insulin secretion, the closure of the ATP-sensitive, potassium channels (K^+^_ATP_ channels) [49].

### K^+^_ATP_ and Voltage-Dependent Ca^2+^ Channels

**Figure 2:**
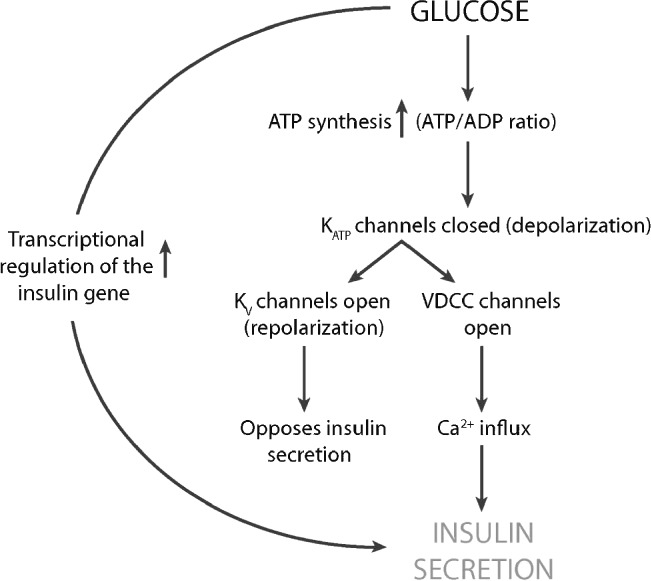
Signalling cascade of first-phase insulin secretion in the β-cell in response to glucose, without the contributions of GLP-1. Glucose metabolism triggers a cellular increase in the ATP/ADP ratio which inhibits K^+^_ATP_ channels leading to depolarization. Depolarization triggers both the opening of the voltage-dependent K^+^ channels (K_v_) to repolarize the β-cell and the activation of VDCCs. The resulting inward Ca^2+^ influx triggers exocytosis of insulin-containing granules leading to insulin secretion.

The major triggering step to insulin secretion is the inactivation of K^+^_ATP_ channels and the resulting depolarization of the β-cell (figure 2). The K^+^_ATP_ channel is made up of four pore-forming K_ir6.2_ subunits and four sulphonylurea receptor (SUR1) regulatory subunits that together regulate pore permissibility. The K_ir6.2_ subunits act as glucose/ATP sensors by binding ATP in a Mg^2+^-dependent manner to undergo a conformational change that closes the channel [50]. In opposition, Mg^2+^-ADP opens the channel by binding to the SUR1 subunits [51]. The action of ATP to close K^+^_ATP_ channels and its counteraction by ADP highlights the important role of glucose metabolism in regulating channel activity. At low glucose concentrations and low ATP/ADP ratios, the channel is open, allowing K^+^ ions to flow out of the cell along their concentration gradient to maintain a hyperpolarized membrane potential of between −65 and −53 mV [48]. However at high glucose concentrations, the ATP/ADP ratio increases leading to channel closure and membrane depolarization. The sulphonylurea family of antidiabetes drugs acts at this step in the pathway. By binding to SUR1 subunits, sulphonylureas such as glimepiride alter the pore conformation causing pore closure and subsequent depolarization of the β-cell independent of ATP/ADP concentrations.

The primary downstream effect of this depolarization event is a change in β-cell membrane potential and activation of L-type voltage-dependent Ca^2+^ channels (VDCC). Depolarization through inactivation of K^+^_ATP_ channels, followed by the ensuing activation of VDCC channels, leads to an influx of Ca^2+^ from the extracellular environment. It is this increase in intracellular Ca^2+^ in the β-cell that facilitates exocytosis, promoting insulin secretion.

### Glucose-Mediated Exocytosis of Insulin

**Figure 3:**
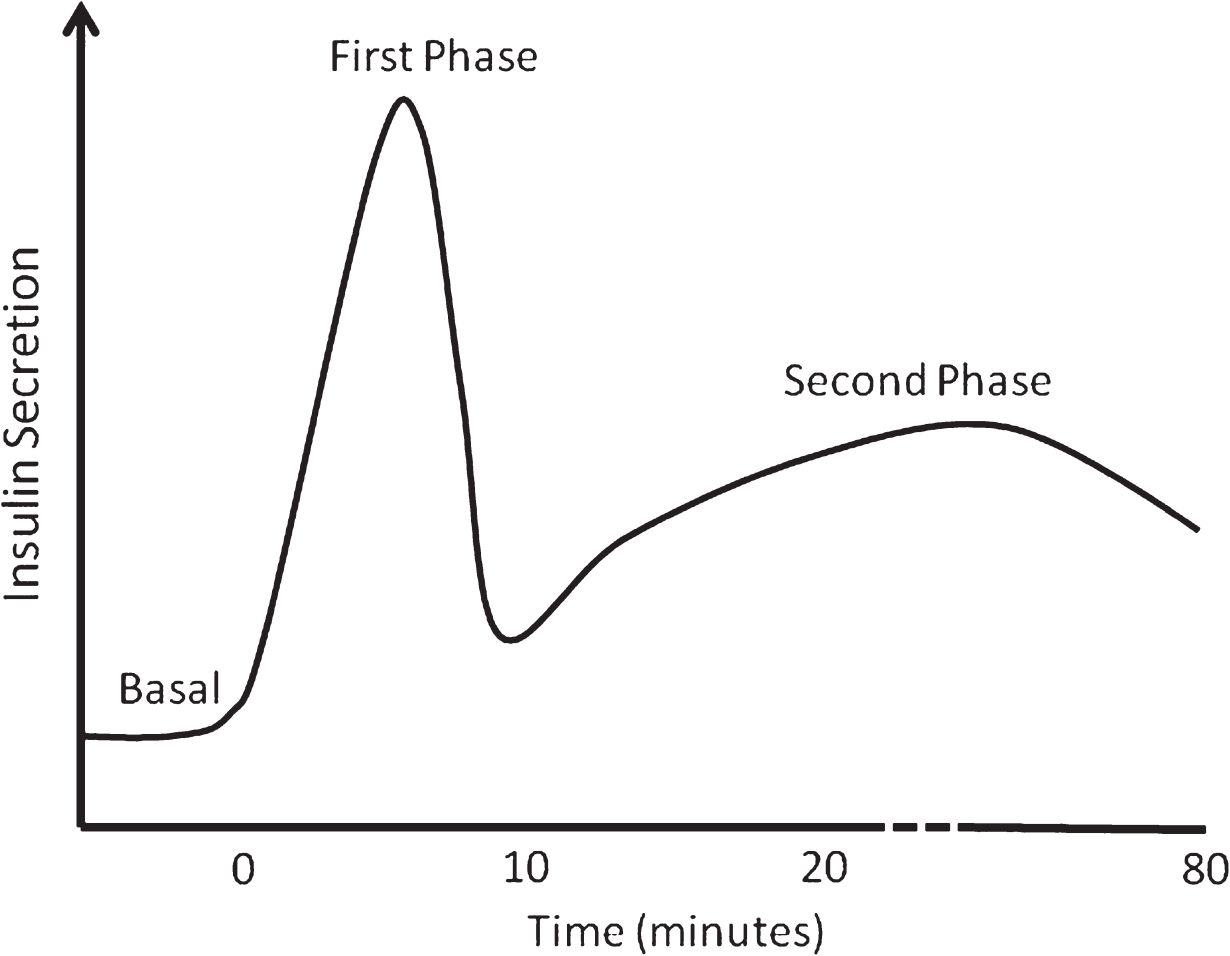
Phases of insulin secretion in response to a square wave of hyperglycaemia. Initial levels of basal insulin production are low. With the induction of hyperglycaemia, a large and rapid insulin secretion occurs that quickly peaks and then falls to levels above basal for an extended period of time.

The insulin hormone itself is synthesized in the endoplasmic reticulum and both stored and transported in large dense core vesicles that become the secretory granules [52]. Insulin is released from pancreatic β-cells via exocytotic processes that are reminiscent of those identified for synaptic vesicles of neuronal cells. However, while the processes of exocytosis are similar to those in neuronal cells, the kinetics of release are quite different. It has been known for some time that insulin secretion in response to glucose occurs in a biphasic pattern with an initial first-phase secretion that is rapid and transient followed by a second-phase of secretion that is slower but sustained at a level above the pre-stimulatory rate (figure 3) [53]. Interestingly, the second phase can only be elicited by secretagogues that generate ATP, suggesting that the second-phase is an energy-dependent process [54].

The process of exocytosis involves the docking and fusion of secretory vesicles to the plasma membrane. This process is mediated by a group of complementary proteins called SNARE proteins [SNAP (soluble NSF attachment protein) receptor]. The vesicle membrane SNARE (v-SNARE) synaptobrevin interacts with plasma membrane SNAREs (t-SNAREs) syntaxin1 and SNAP 25 to form a stable complex in close proximity with the plasma membrane, positioning it for membrane fusion [53,55]. Fusion of the vesicular membrane to the plasma membrane is necessary for the secretion of insulin from the β-cell. While the exocytosis process is well known to be regulated by Ca^2+^, the exact mechanisms by which Ca^2+^ triggers exocytosis through the SNARE protein complex has not been fully elucidated.

It has been estimated that approximately 50–200 of the β-cell's 10,000 insulin-containing granules are released in the rapid, first phase of insulin release [56–58]. This is in contrast to a release rate of 5–40 granules/min from a single β-cell during the prolonged second phase of insulin secretion [56]. Given that the second phase can persist for up to several hours depending on glucose blood levels, the second phase of insulin secretion can contribute significantly to the overall insulin release [56–60].

**Figure 4:**
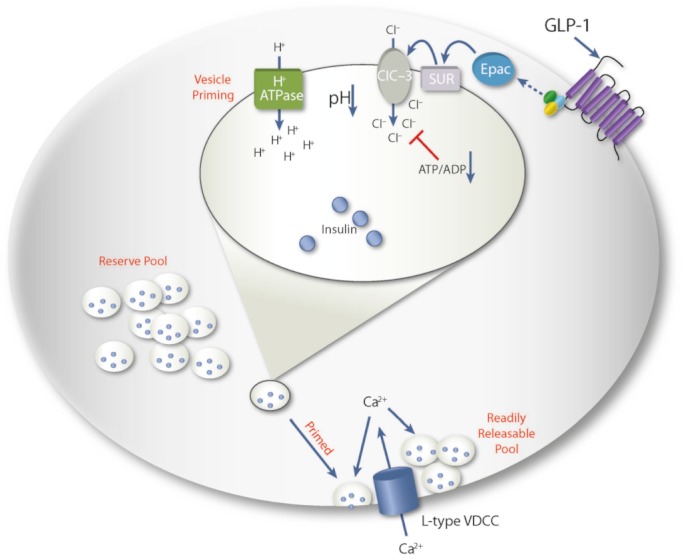
Putative vesicle priming events that allow vesicles to become competent for exocytosis. Once primed, vesicles ‘move’ from the reserve pool to the readily releasable pool where the increase in Ca^2+^ influx through activated VDCCs promotes exocytosis. GLP-1 may promote Cl^-^ ion pumping into a vesicle through Epac and a putative granule SUR protein that may regulate ClC-3 chloride channels. The influx of Cl^-^ ions counters the charge promoted by the action of the H^+^-ATPase pump. The increase in H^+^ allows the decrease in intragranular pH necessary for exocytosis.

The rate at which insulin can be secreted depends largely on the number and competency of the insulin containing granules available. It is thought that the granules exist in distinct pools within the β-cell, in either a readily-releasable pool or a reserve pool (figure 4). The difference between the two pools is the releasability of the granule contents. Only granules that are ‘primed’ for exocytosis will release their contents in response to Ca^2+^ signals. Most of the insulin granules (∼95–99%) belong to the reserve pool while a smaller fraction (only ∼1–5%) belong to the readily releasable pool that is thought to reside closer to the plasma membrane than the reserve pool [54]. The readily-releasable pool is further subdivided into a third pool of granules, the immediately-releasable pool, which is physically docked at the plasma membrane in association with VDCC and primed for immediate release of insulin upon VDCC channel opening and initial Ca^2+^ entry [49]. The release of the contents of the readily releasable pool is largely responsible for the first-phase of insulin secretion while the slower second-phase reflects the refilling of the readily-releasable pool via mobilization and priming of granules from the reserve pool [60].

In order for granules from the reserve pool to become competent for release, they must ‘move to’ the readily-releasable pool (figure 4). Acidification of the insulin granule is one of the steps in this process. Although the precise role of granule acidification in priming granules for exocytosis is unknown, it is hypothesized that a low granular pH may induce a conformational change in the SNARE proteins that facilitate membrane fusion [56].

Granule acidification occurs through V-type H^+^-ATPases that facilitate H^+^-pumping into the granules, reducing the intragranular pH. In concert with the H^+^-ATPase, Cl^−^ channels localized to the granules (ClC-3) also play an essential role in granule acidification by allowing influx of Cl^−^ ions [59]. Without the entry of the Cl^−^ counter ion, a large electrical gradient would develop from the accumulation of H^+^ ions, halting further H^+^ pumping and preventing acidification [59,61]. That acidification of granules is required for Ca^2+^ induced exocytosis is suggested by the finding that exocytosis is inhibited by protonophores or inhibitors of the vesicular H^+^ pump [59]. Similarly, depolarization-evoked exocytosis is decreased in β-cells of ClC-3 knockout mice and by Cl^−^ channel blockage with DIDS (4, 4′-diisothiocyanatostilbene-2, 2′-disulfonic acid) [59,62].

### Glucose-Mediated Transcription of the Insulin Gene

Only a small portion of the total intracellular insulin pool is released during glucose-mediated insulin secretion, thus insulin availability depends more upon the regulation of secretion than on the rate of insulin biosynthesis [52]. Nonetheless, it has been demonstrated in foetal rat islets that the insulin transcriptional promoter is glucose-responsive, inducing gene expression almost seven-fold in response to 16 mM glucose [63]. Further, glucose has been shown to increase the stability of insulin mRNA [64]. Accordingly, in addition to promoting the secretion of premade and stored insulin, glucose also acts to ensure that appropriate amounts of insulin are available when needed.

## GLP-1 Receptor Signalling Augments Glucose-Mediated Insulin Release

### The Relationship of GLP-1 and Glucose

**Figure 5:**
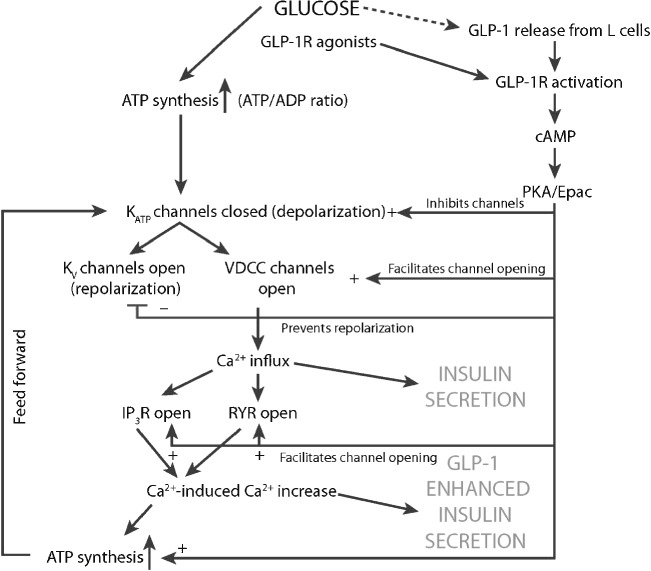
Signalling cascade of insulin secretion in the β-cells in response to glucose and GLP-1. Glucose triggers the cascade of events outlined in figure 1. GLP-1 potentiates the activity of glucose by inhibiting K^+^_ATP_ channels, facilitating the opening of VDCCs, and inhibiting membrane repolarization via K_v_ channels. GLP-1 also sensitizes IP_3_ (IP_3_R) and ryanodine (RYR) receptors to the effects of Ca^2+^, facilitating Ca^2+^-induced Ca^2+^ release (CICR). CICR promotes enhanced insulin secretion and enhanced ATP production, the latter of which facilitates a feed-forward loop to promote further rounds of depolarization and insulin secretion.

In addition to triggering insulin secretion directly through the above-described series of events, ingested glucose also stimulates the transcription and release of the insulin secretagogue, GLP-1 (figure 5) [65]. Orally-consumed carbohydrates, proteins, and lipids induce the secretion of GLP-1 from intestinal L-cells; however, GLP-1-mediated stimulation of insulin secretion from β-cells is only observed in the presence of elevated glucose concentrations.

### cAMP Production

**Figure 6:**
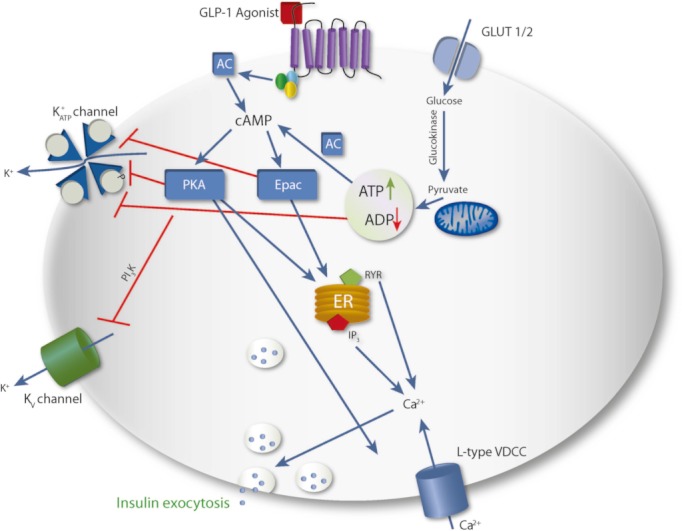
A schematic overview of the main signalling cascades involved in insulin secretion. Glucose enters the β-cell through the GLUT1/2 transporters and is converted to ATP. GLP-1 secreted from L-cells in response to glucose and other nutrients, binds to the GLP-1 receptor, triggering an increase in cAMP and activation of Epac and PKA; cAMP is also produced from soluble adenylate cyclase adding to the overall concentration of cAMP. Glucose metabolism leads to an increase in ATP/ADP concentrations which act to inhibit K_ATP_ channels. The inability to pump K^+^ from the β-cell leads to membrane depolarization and opening of VDCC. GLP-1-activated PKA and Epac potentiate the effects of glucose by further inhibiting both the K^+^_ATP_ and the K_v_ channels, preventing repolarization of the β-cell. PKA and Epac also sensitize multi-subunit calcium channels on the endoplasmic reticulum (ER) allowing the release of calcium from intracellular stores. This increase in free calcium concentration further aids in exocytosis of insulin.

GLP-1 signalling is initially transduced through binding to and activation of the GLP-1R found on the β-cells of the pancreas (figure 6). The GLP-1R is a ligand-specific, seven-transmembrane domain, G-protein coupled receptor (GPCR). When activated by ligand binding, the GLP-1R couples to the trimeric G-protein complex and facilitates the release of the activated Gα_s_ subunit of the complex which then, in turn, activates plasma membrane-bound adenylyl cyclase (AC) to produce cAMP [48].

As it is synthesized from ATP, cAMP, the primary second messenger of the GLP-1R is necessarily dependent on the availability of the product of glucose metabolism, ATP. However, the question of whether increases in cellular ATP lead to increased cAMP production is controversial [66,67]. As euglycaemia is not a requirement for all cAMP-regulated signalling, it is unlikely that increases in cAMP due to increases in ATP concentration play a role in the glucose dependence of GLP-1R-mediated insulin secretion.

As is common for GPCR biology, the GLP-1R activates plasma membrane-bound AC (tmAC; transmembrane AC) leading to rapid cAMP production. However, other distinct ACs of the soluble AC (sAC) family have also been shown to contribute to the overall concentration of cAMP in the presence of high glucose concentrations, albeit with slower kinetics than with the GLP-1R [68]. It is possible that, in the presence of elevated glucose concentrations, the combined activity of the tmAC and the sAC may potentiate GLP-1R-induced insulin secretion through higher cellular cAMP levels.

### Consequences of cAMP Generation

The two primary downstream effectors of GLP-1R-induced cAMP are protein kinase A (PKA) and the cAMP-regulated guanine nucleotide exchange factor (cAMP-GEF), Epac. That PKA and Epac each play critical roles in GLP-1-mediated insulin release is evidenced by the ability of Epac or PKA specific activators (or inhibitors) to potentiate (or inhibit) glucose-dependent insulin secretion [69–72]. Thus, the activity of the GLP-1R is transmitted through the actions of PKA and Epac.

PKA is a holoenzyme composed of regulatory subunits and catalytic subunits. The binding of cAMP to the regulatory subunits releases the catalytic subunits which then act to phosphorylate downstream substrates. The single polypeptide protein, Epac, is similarly activated by cAMP. Upon cAMP binding, Epac undergoes a conformational change that prevents the interaction between its regulatory and catalytic domains. Epac primarily functions as a guanine nucleotide exchange factor for small Ras-like G-proteins, thereby regulating their activity; however, direct Epac protein/protein interactions have also been identified [13,48,73].

### Convergence of Glucose-Stimulated and GLP-1R-Stimulated Insulin Secretion

As mentioned previously, the initial triggering step in glucose-mediated insulin secretion is the inhibition of K^+^_ATP_ channels and depolarization of the β-cell. Glucose and GLP-1R agonist activities cooperatively converge at this initial step. As discussed above, the K_ir6.2_ subunits of the K^+^_ATP_ channel bind ATP, facilitating channel closure and depolarization of the cell while the SUR1 subunits counter channel closure by binding ADP, promoting channel permeability. Phosphorylation of the SUR1 subunits by one of the GLP-1R effector proteins, PKA, disrupts the binding of ADP thereby tipping the balance of pore regulation towards channel closure and depolarization of the cell [67,74]. The coordinated actions of channel inhibition through glucose metabolism and the inhibition of channel activation via phosphorylation of SUR1 subunits lead to a synergistic closure of K^+^_ATP_ channels. Interestingly, in addition to providing synergistic assistance in K^+^_ATP_ channel closure, the activity of PKA to regulate K^+^_ATP_ channels is also truly glucose-dependent [67]. Studies by Light et al. suggest that K^+^_ATP_ channel closure via PKA is dependent on reduced ADP concentrations as occurs under conditions of high glucose; at elevated ADP levels, as in times of low glucose, the effect of PKA on channel closure is negligible [48,67]. The glucose dependence of this critical triggering step prevents GLP-1R agonists from potentially initiating β-cell depolarization in the absence of glucose.

The other key regulator of GLP-1R activity, Epac, also modulates K^+^_ATP_ channels. Experiments using patches of plasma membrane from human β-cells or rat insulinoma cell lines (INS-1) demonstrated that an Epac-selective cAMP analogue significantly reduced the concentration of ATP necessary for 50% inhibition of K^+^_ATP_ channels [75]. These experiments suggest that Epac enhances channel inhibition by increasing the effectiveness of ATP on channel closure [10,75]. Consequently activities of the GLP-1R effectors, PKA to inhibit channel activation (through ADP binding), and Epac to facilitate channel inhibition (through ATP binding), result in a coordinated potentiation of glucose-initiated cell membrane depolarization. Together, these activities facilitate the next step in the cascade towards insulin secretion, the opening of VDCC.

In addition to aiding in K^+^_ATP_ channel inhibition, GLP-1R signalling also inhibits a compensatory repolarization event mediated by the opening of voltage-dependent K^+^ channels (K_v_). In response to membrane depolarization, voltage-dependent K^+^ channels open to restore the resting membrane potential through an increase in the outward flow of K^+^ ions. GLP-1R agonists (GLP-1 and exendin-4) opposed this efflux of K^+^ as demonstrated by experiments conducted in rat β-cells which showed an inhibitory effect of exendin-4 on voltage-dependent outward K^+^ currents [76]. This inhibition of K^+^ efflux delays membrane repolarization, allowing a longer period of Ca^2+^ influx through VDCCs and ultimately greater insulin secretion [77,78].

### Effects of GLP-1 on Voltage-Dependent Ca^2+^ Channels

Data from several groups has suggested that GLP-1 also potentiates glucose-mediated insulin secretion by increasing the magnitude of the inward Ca^2+^ current produced by VDCCs [49,79–81]. This effect is suggested to be PKA-dependent as PKA inhibition blocked GLP-1-mediated augmentation of the Ca^2+^ current [81]. Phosphorylation of channel components by PKA is likely responsible for modulating pore activity [49,82,83]. Accordingly, as glucose activates VDCCs through β-cell depolarization, GLP-1 potentiates this activity by further altering the permeability of the VDCC, allowing greater influx of Ca^2+^.

### GLP-1 and Ca^2+^ Induced Ca^2+^ Release

Once Ca^2+^ enters the β-cell, it plays several roles in insulin secretion. One role, as mentioned previously, is in the initiation of exocytosis of insulin via membrane fusion of the readily-releasable granules during the first phase of insulin secretion. A second role of Ca^2+^ is in a Ca^2+^-induced release (CICR) of more Ca^2+^ from intracellular stores in the endoplasmic reticulum [84,85] and secretory granules [85,86]. This increase in free intracellular Ca^2+^ from intracellular stores permits enhanced granule exocytosis leading to enhanced insulin secretion.

The inositol triphosphate receptor (IP_3_R) and the ryanodine receptor (RYR) control this release of Ca^2+^ from intracellular stores and antagonists to these receptors inhibit CICR [87]. These two receptor families are multi-subunit Ca^2+^ channels that alternate between conformations that permit or inhibit the release of Ca^2+^ from intracellular stores [88,89]. The regulatory domain of the IP_3_R contains binding sites for Ca^2+^ that control the activity of the channel dependent in part on free Ca^2+^ concentration [88]. Likewise, the RYR is similarly regulated by Ca^2+^ binding to cytosolic sites on the complex. GLP-1R agonists promote CICR in a Ca^2+^-dependent manner, requiring a rapid increase in intracellular free Ca^2+^ concentration to mediate Ca^2+^ release from intracellular stores [85,89]. Indeed, exendin-4 is unable to induce CICR in INS-1 cells in the presence of caged Ca^2+^ (calcium that is not free) but potently induces CICR when Ca^2+^ is uncaged [87]. Endogenously, the rapid Ca^2+^ increase required for GLP-1R-stimulated CICR can be provided by glucose-induced VDCC opening. This hypothesis is supported by the finding that GLP-1 induced CICR does not occur in the presence of a VDCC-inhibitor, nifedipine [49]. In this manner, GLP-1R-promoted CICR is dependent upon glucose-initiated events and cannot mediate enhanced insulin secretion without the downstream function of glucose.

GLP-1R activation participates in CICR by sensitizing the intracellular Ca^2+^channels (IP_3_R and RYR) to the stimulatory effects of Ca^2+^. This cAMP-dependent effect is mediated via both PKA and Epac [48,90] and cAMP analogues that activate both PKA and Epac can substitute for GLP-1 by inducing CICR in the presence of Ca^2+^ [87]. The use of specific cAMP analogues and ryanodine or IP_3_ receptor antagonists demonstrated that Epac sensitizes the ryanodine receptors and PKA the IP_3_ receptors [85,87,91].

In addition to increasing the free intracellular Ca^2+^ that triggers enhanced insulin exocytosis, GLP-1R-stimulated CICR also facilitates the production of ATP. Using dynamic bioluminescence imaging to record ATP and Ca^2+^concentrations, Tsuboi et al. detected GLP-1-evoked increases in both Ca^2+^ and ATP concentration. However, chelation of intracellular Ca^2+^ inhibited the ability of GLP-1 to increase ATP concentrations, suggesting that CICR is essential for elevation of ATP by GLP-1 [91]. The group proposed that increased Ca^2+^ activates mitochondrial dehydrogenases (i.e. pyruvate, isocitrate and 2-oxoglutarate dehydrogenases [48]) to promote increased ATP production in the presence of glucose [91]. This production of ATP and modulation of the cellular ATP/ADP ratio may lead to continued closure of K^+^_ATP_ channels and further depolarization. In this manner, GLP-1R stimulation promotes continued insulin secretion until extracellular glucose concentrations fall in response to insulin.

### Granule Recruitment and Replenishment

The activation of PKA through the GLP-1R not only facilitates the secretion of insulin contained in granules competent for exocytosis, but also modulates the refilling of the readily-releasable pools, an event important for the second phase of insulin secretion [49,92]. Using perforated-patch whole-cell recordings of mouse β-cells, intense trains of depolarizations were repeated to induce Ca^2+^ influx leading to exocytosis. Over time, Ca^2+^ influx eventually failed to produce exocytosis suggesting that the readily-releasable pool can be depleted. Sustained induction of cAMP by forskolin allowed depolarization-promoted exocytosis to continue significantly longer, suggesting that cAMP accelerates the mobilization or priming of vesicles from the reserve pool into the release-competent readily-releasable pool [92]. PKA inhibitors only partially counteracted this effect suggesting that the action of cAMP to promote Ca^2+^-dependent exocytosis has both PKA-dependent and independent components.

Further studies examining the PKA-independent component of Ca^2+^-induced exocytosis suggested that part of the cAMP-mediated activity was Epac-dependent. In murine β-cells, exocytosis mediated by a train of depolarizations could be increased by the selective Epac agonist 8CPT-2Me-cAMP. This effect was most pronounced during early exocytosis suggesting that Epac may increase the size of the immediately-releasable/readily-releasable pools that rapidly respond to Ca^2+^ influx [93]. The combined effects of Epac and PKA to promote both larger releasable insulin pools and the refilling of depleted pools are consistent with the observed increases in first and second phase insulin secretion in response to GLP-1R activation.

In addition to assisting with the release of already stored insulin, GLP-1 also acts to replenish the insulin hormone after secretion by inducing transcription from the insulin gene in a CREB-, CREM-, and ATF-1-independent manner [94].

### Promotion of Vesicle Priming

Both the increase in the readily-releasable insulin pool size and enhanced insulin pool refilling depend upon the ability of GLP-1 to promote priming events necessary to allow granules from the releasable pool to ‘move to’ the readily-releasable pool. Granule priming is a multi-step process requiring ATP, Ca^2+^, and undefined temperature-dependent events [56]. Although the mechanisms are far from clear, granule acidification is a metabolically regulated priming step that may be modulated by GLP-1R effectors.

As described previously, acidification of insulin granules requires H^+^-ATPases that facilitate H^+^ pumping into the granules and Cl^−^ channels that allow entry of the Cl^−^ counter ion. The influx of Cl^−^ ions may be endogenously regulated by the actions of SUR1 localized to granules. The influx of Cl^-^ ions was observed to be accelerated by cAMP and this effect was abolished in SUR1-deficient β-cells [93]. Supported by the observation of a direct interaction of Epac with SUR1 [73], cAMP may accelerate Cl^−^ influx via Epac acting on granular SUR1 (figure 4). Consistent with the finding that Epac enhances granule pool size, increased influx of Cl^−^ may facilitate granule acidification and priming, thereby increasing the insulin pool size [48,72,93]. Indeed, the Epac-dependent augmentation of early exocytosis (resulting from increased pool size) was diminished in β-cells from SUR1-deficient mice [93].

The step of granule acidification may additionally be glucose-regulated. By monitoring granule pH, it was observed that ADP abolished granule acidification (even in the presence of ATP) much like a Cl^−^ channel blocker. The authors suggested that ADP might act to inhibit acidification by directly or indirectly inhibiting granular Cl^−^ uptake through the Cl^−^ channel [59]. Thus, at times of low glucose, ADP would inhibit granule acidification and vesicle priming, thereby preventing GLP-1R agonist-mediated enhancement of early insulin pools or the recruitment of reserve pools.

### The Interaction of GLP-1 and Sulphonylureas

The role of the SUR1 receptor in first-phase insulin secretion, and potentially in granule acidification required for second-phase secretion, provides a possible explanation for the observed clinical interaction between GLP-1R agonists and SUs. Several human clinical trials have demonstrated that patients using GLP-1R agonists and SU co-therapies have a greater incidence of hypoglycaemia than those not using SU (Table 1) [18,19,44,45,95]. It is possible that co-treatment with an SU and a GLP-1R agonist overrides the inherent ability of the GLP-1R agonist to minimize hypoglycaemia. Indeed, SU-based compounds have been shown to uncouple the glucose dependence of GLP-1R agonist activity in the rat pancreas [96]. This uncoupling effect is additionally suggested by a clinical study in Japanese patients with T2D in which significantly fewer patients treated with placebo on a background SU therapy (with or without other oral therapies) experienced hypoglycaemia than patients treated with exenatide BID and background SU (10 and 54%, respectively; p < 0.001)[97]. Although the mechanism of this proposed uncoupling event has not been fully elucidated, our understanding of the GLP-1R signalling cascade suggests that SUs may allow GLP-1R agonists to bypass glucose dependence by triggering β-cell depolarization even in the absence of glucose. Thus, SUs that bind SUR1 to modulate K^+^_ATP_ channels independently of glucose may also allow GLP-1R agonists to bypass their inherent glucose requirement by stimulating the downstream effects ordinarily associated with increased glucose.

### Limitations of Current Knowledge

Although studied for decades, our understanding of the cascade of events beginning at glucose stimulation and leading to insulin secretion remains to be fully understood. The molecular mechanisms of glucose-stimulated first-phase insulin secretion and the role of GLP-1 in the potentiation of glucose-mediated events have been characterized to a much greater extent than mechanisms involved in second-phase insulin secretion. Currently, the accepted model of insulin secretion is that of a separate first and second-phase of insulin secretion due to the release of insulin from functionally distinct and regulatable pools [54]. However, much remains to be elucidated regarding the timing, priming, localization, mobilization and membrane fusion of the insulin granules as well as the proteins involved in these events.

## Summary

The treatment of T2D focuses on glucose control, which is essentially the reduction of hyperglycaemia while minimizing hypoglycaemia. Although many diabetes therapies successfully manage the hyperglycaemia, non-self-regulating therapies occasionally tip the balance towards hypoglycaemia. Hypoglycaemia carries with it several undesirable acute and chronic outcomes and its avoidance is an important clinical concern. Comparisons between the GLP-1R agonists and other insulinotropic drugs such as insulin glargine or the sulphonylurea, glimepiride, have demonstrated that, in addition to better reduction in HbA1c, patients using GLP-1R agonists experience a significantly lower incidence of overall hypoglycaemia, [18,20,23,42] consistent with the glucose-dependent nature of GLP-1R agonists. The glucose dependence of the GLP-1R agonist class of T2D therapies provides an intrinsic measure of glycaemic control. As self-regulating therapies, they possess built-in monitors of blood glucose, signalling to boost insulin secretion at times of high glucose and halting action as glucose levels fall below euglycaemic levels in response to insulin. In this manner, GLP-1R agonist therapies provide glucose lowering effects with the additional benefit of a lower limit for glucose concentrations, consistent with glycaemic safety.

The insulinotropic action of GLP-1R agonists has been shown *in vitro* and *in vivo* to depend on β-cell detection of elevated glucose concentrations (above approximately 3.9 mmol/l). Indeed, although the basic GPCR activation cascades are likely activated by GLP-1 ligand binding to its receptor, several signalling blockades halt the message prior to insulin secretion in the absence of glucose. The most important of these blocks occurs at the K^+^_ATP_ channel where GLP-1 promotes PKA-mediated phosphorylation of the SUR1 subunit in a glucose-dependent manner. Increased ADP concentrations prevent SUR1 phosphorylation by PKA, repressing the effect of GLP-1 at this important step. Similarly, it is speculated that increased ADP concentrations may also inhibit GLP-1-mediated granule acidification and thus prevent vesicle priming events.

Several steps in the cascade are also glucose-dependent by virtue of the fact that the GLP-1-mediated events cannot occur in the absence of earlier, glucose-mediated events. For example, both the GLP-1-mediated augmentation of the Ca^2+^current through the VDCC and the GLP-1-mediated induction of CICR first require the activation of the VDCC, an event that occurs as a result of glucose-stimulated, β-cell depolarization. By facilitating glucose-initiated signalling events at many steps along the path toward insulin secretion, GLP-1 and, similarly, GLP-1R agonists act as enhancers of glucose-induced activities and have little to no effect on insulin secretion in the absence of glucose. Accordingly, GLP-1R agonists, known to possess glucose-dependent activity, can also be seen as synergistic partners of glucose during glucose-stimulated insulin secretion.

Glucose in the body is controlled by an intrinsically balanced system of overlapping and interconnected signalling pathways such that signalling from the GLP-1R both regulates glucose and is regulated by glucose. For patients with diabetes, GLP-1R agonists have been shown to improve glycaemic control in a regulated manner; lowering elevated blood glucose while maintaining sufficient concentrations for optimal metabolic function.
